# Health Care Provider Perspectives on the Use of a Digital Behavioral Health App to Support Patients: Qualitative Study

**DOI:** 10.2196/28538

**Published:** 2021-09-28

**Authors:** Valerie Silfee, Kelly Williams, Brett Leber, Jane Kogan, Cara Nikolajski, Eva Szigethy, Catherine Serio

**Affiliations:** 1 UPMC Health Plan Pittsburgh, PA United States; 2 UPMC Center for High-Value Health Care Pittsburgh, PA United States; 3 Department of Psychiatry University of Pittsburgh Pittsburgh, PA United States

**Keywords:** digital health, mHealth, implementation, cognitive behavioral therapy, anxiety, depression, smartphone, mobile phone

## Abstract

**Background:**

Despite the growing evidence indicating the efficacy of digital cognitive behavioral interventions (dCBIs) for behavioral health (BH) treatment, broad and consistent use of such interventions has been limited by knowledge obtained in real-world settings, including factors that impact provider uptake/referral. Engaging providers early in the implementation process offers an opportunity to explore their needs and behaviors, integrate interventions into workflows, and better understand provider setting capabilities.

**Objective:**

This study assessed providers’ views on the feasibility and acceptability of delivering a cognitive behavioral therapy (CBT)-based mobile app in multiple care settings.

**Methods:**

Participating providers included BH and physical health (PH) providers from a women’s health center, an outpatient BH clinic, and both rural/urban primary care settings. All participating providers cocreated workflows through facilitated workshops, including establishing feedback loops between the project team and providers and identifying clinical champions at each site. Over a 12-week period, the providers referred adult patients experiencing anxiety or depression to a mobile app-based dCBI, RxWell, and provided other indicated treatments as part of usual care. Referrals were completed by the providers through the electronic medical record. To better understand facilitators of and challenges in integrating RxWell into routine practice and perceptions of sustainability, a series of qualitative interviews was conducted. Interview data were analyzed to identify major themes using an inductive content analysis approach.

**Results:**

A total of 19 provider interviews were conducted to discover motivators and barriers for referring RxWell. The providers benefited from a focused discussion on how to incorporate the referral process into their workflow, and knowing the app content was rooted in evidence. Although the providers believed engaging in experiential learning was important, they indicated that more education on the digital health coach role and how to monitor patient progress is needed. The providers thought patient engagement may be impacted by motivation, a lack of comfort using a smartphone, or preference for in-person therapy. The providers also expressed enthusiasm in continuing to refer the app. They liked the ability to provide patients with support between sessions, to have an extra treatment option that teaches BH exercises, and to have a CBT treatment option that overcomes barriers (eg, wait times, copays, travel) to traditional therapy modalities.

**Conclusions:**

Digital intervention success in health care settings relies heavily on engagement of key stakeholders, such as providers, in both design and implementation of the intervention and focused evaluation within intended care setting(s). Scaling digital interventions to meet the mental health needs of patients in usual care settings leans on thoughtfully constructed and streamlined workflows to enable seamless referral of patients by providers. Our findings strongly suggest that providers are supportive of digital tool integration to support the mental health of patients and endorse its use within their routine workflow.

## Introduction

Approximately 1 in 5 adults in the United States lives with a mental illness [1], with 40 million affected by anxiety and 16 million affected by depression annually [2,3]. Coronavirus disease 2019 (COVID-19) has intensified symptom expression, with more than half of Americans reporting worsening mental health due to COVID-19 and 1 in 5 anticipating the pandemic to majorly impact their overall functioning [4]. Although effective treatments are available to manage anxiety and depression, including medication [5] and psychotherapy (including cognitive behavioral therapy [CBT]) [6-8], many barriers exist in providing and receiving mental health care, leading to less than half of those experiencing mental health issues receiving treatment [1].

Barriers to receiving mental health care include stigma [9,10], high costs for care [11], limits in health insurance access or in-network care [12], and limited access to mental health care professionals [13]. There are shortages of mental health professionals across the United States, with data from 2018 estimating that only one-quarter of the nation’s need for a mental health provider are being met [12]. Provider shortages leave patients with extended wait times and longer distances to travel for care [12], with 1 study finding patients waiting for up to 90 days for an initial psychiatric visit [14]. Moreover, two-thirds of primary care physicians (PCPs) struggle to obtain timely outpatient mental health services for patients, citing lack of mental health care providers, health plan barriers, and inadequate health insurance coverage as important challenges [15]. PCPs often serve as the main source of treatment for patients with common mental health concerns, and workforce shortages in primary care settings present yet another barrier to accessing mental health care [16].

Digital cognitive behavioral interventions (dCBIs) can address barriers and care gaps in mental health care by increasing treatment accessibility and reducing the time it takes to receive care. It is estimated that over 10,000 of the 325,000 commercially available health and wellness digital apps specifically target mental or behavioral health (BH) [17], and over the past several years, significant investments of up to $9.4 billion in the United Sates (2020) have been made to develop, validate, and regulate these apps for clinical use [18,19]. Undoubtedly, the demands of COVID-19 on our health care system have accelerated the opportunity to improve access to mental health services through digital health technologies [20].

There is a growing interest among providers to have access to digital tools [21]. Mobile app–based dCBIs have the potential to effectively reduce stress, anxiety, and depression [22-25], and improve care delivery for providers seeking to scale their mental health treatment options, streamline their workload, improve patient experience, and lower cost [26,27]. Unfortunately, providers are infrequently referring and encouraging the use of a dCBI in their practices due to barriers such as a lack of integrated workflows (eg, prescribing and monitoring through the electronic medical record), low confidence in the app content or security, high costs, and limited bandwidth to identify, from thousands of commercial apps, the right one for their patients [18,28,29].

There are many factors that influence the uptake and scalability of dCBIs in provider settings. Engaging providers early in the implementation process offers an opportunity to explore their needs and behaviors, integrate interventions into workflows, and better understand capabilities and limitations of routine care settings [30,31]. Learning more about provider perspectives is an important step in addressing the complex issues around the effective implementation and scalability of digital tools [17,31,32]. Our study provides results from a system-level quality improvement initiative that includes an assessment of providers’ views on the feasibility and acceptability of delivering a dCBI, RxWell, in multiple routine care settings.

## Methods

### Quality Improvement Initiative Overview

UPMC is a large integrated delivery and finance system (IDFS) headquartered in Western Pennsylvania, offering both medical care and health care coverage to more than 3.9 million members, while supporting and improving care across the continuum [33]. Participating care settings were identified from the UPMC provider network based on interest in participating in a quality improvement initiative focused on improving uptake of RxWell, a dCBI developed by UPMC Health Plan. Partnering provider sites included both behavioral and physical health (PH) providers: 1 women’s health center with integrated BH, 1 outpatient BH clinic, and 2 rural/urban primary care and family medicine practices. Over a 12-week period, the providers referred adult patients experiencing depression or anxiety to RxWell, while providing other indicated treatments (medication/therapy referrals) as part of usual care. Referrals were completed by the providers through the electronic medical record. The app was offered to patients at no additional cost. A series of interviews was conducted with both behavioral (eg, clinical psychologist) and physical (eg, PCP) health care providers across 4 health care settings at the conclusion of the initiative. This quality improvement project was approved by the UPMC Quality Improvement Review Committee.

### Implementation Sites: Engagement, Support, and App Referrals

As part of an IDFS, UPMC integrates more than 40 hospitals and 800 doctors’ offices and outpatient sites. These connections supported a targeted approach to clinical site identification based on senior leadership support and site needs for BH support. Initial in-person visits to each care setting were conducted to learn more about provider needs and the capacity to participate as well as to share an overview of RxWell and the evaluation design/plan. Subsequently, all participating partner sites engaged in a facilitated workshop to receive instructional and educational training materials as well as generate and align on a revised clinical workflow that integrated RxWell. Collaborative decisions included the timing and manner of how the app would be introduced and referred to patients, the degree to which providers would follow up on patient progress, and the feedback loop between clinical, research, and coaching staff. Although feedback loop processes varied by site, communication modalities remained consistent (eg, email and phone calls). During these conversations, a gatekeeper/physician champion was identified at each site to support communication throughout the quality improvement project period.

Across all project sites, we engaged 56 providers to refer RxWell. During the project period, a total of 449 patients were referred to RxWell and 164 individuals enrolled in the app, 63 from BH providers and 101 from PH providers. Referring providers had access to a dashboard in the electronic medical record that enabled them to track individual patient progress, including technique completion, number of messages to the digital health coach, and anxiety and depression scores. In addition, the project team emailed aggregate reports to providers at a cadence based on site preference that detailed the number of app referrals and downloads by the provider/site, as well as de-identified patient-level engagement and anxiety/depression score data.

### Digital BH Mobile App

RxWell was developed from evidence-based approaches to BH: CBT and mindfulness. The app combines these approaches with the support of a digital health coach to deliver anxiety or depression programming to patients [34]. The dCBI was designed to be used on its own, as part of a stepped care model, or alongside face-to-face therapy to extend the reach of care, support skill practice, and monitor outcomes. Key app components include audio- and text-based techniques, a digital health coach, and assessment of anxiety/depression symptoms. Patients chose which program to enroll in, with guidance from their provider being available, and had access to RxWell for 6 months. The anxiety and depression programs include 53 and 40 techniques, respectively, and patients can complete up to 5 new techniques per day. See Table 1 for a sample of technique descriptions. In addition, RxWell use metrics are collected through a secure app database, including the number of referrals, number of patients enrolled, patient demographics (age, gender), technique completion, coach messages, and anxiety and depression scores measured by the Generalized Anxiety Disorder 7-item scale (GAD-7) [35] and the Patient Health Questionnaire 8-item scale (PHQ-8) [36].

**Figure 1:**
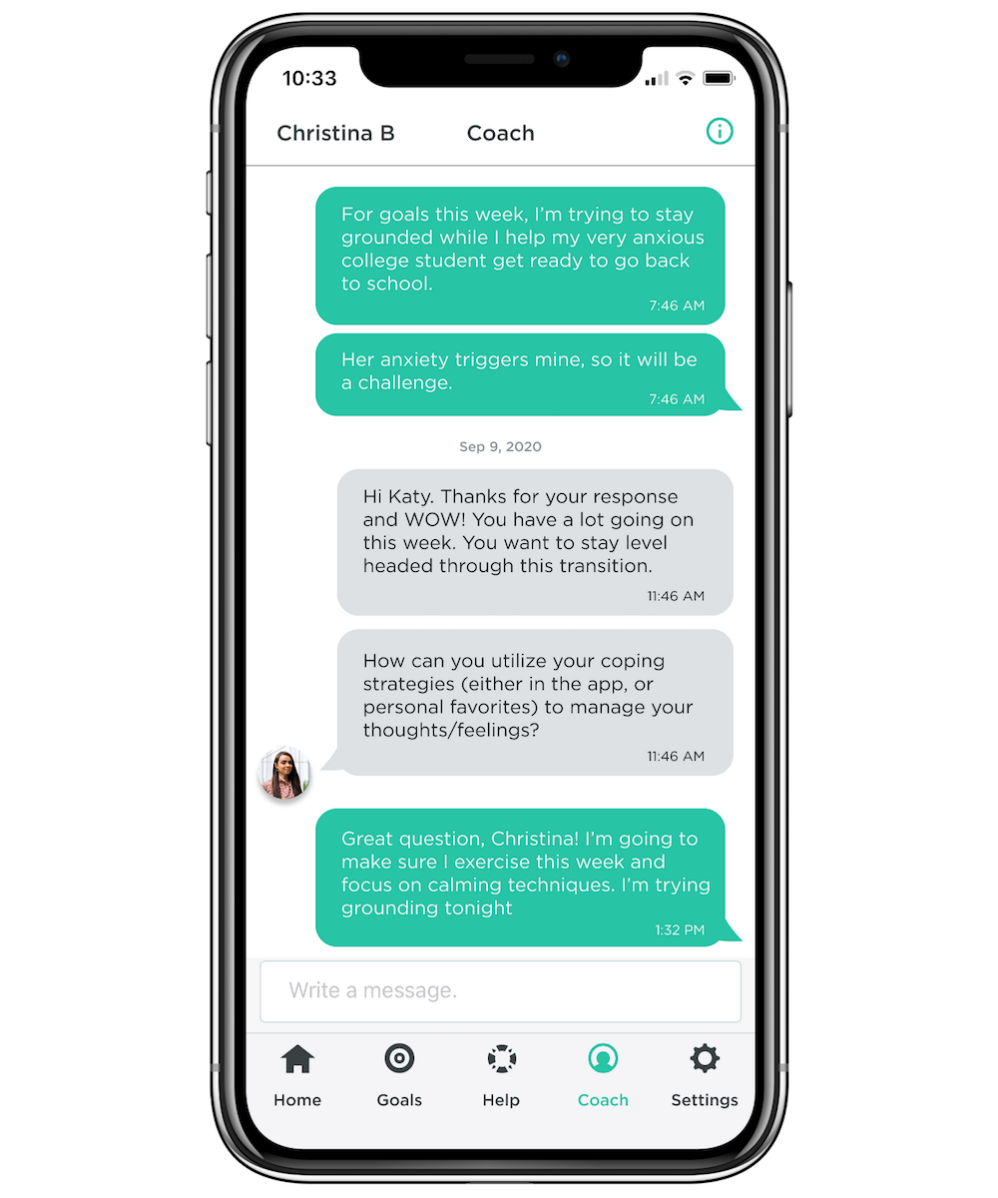
Example interaction between a digital health coach and a user in the app.

Digital health coaches, employed by UPMC, provide in-app support by guiding patients through setting goals, building intrinsic motivation, and recognizing successes. Coaches are college graduates, trained in CBT, mindfulness, and motivational interviewing and supervised by a licensed mental health care provider. All communication between patients and health coaches occurs via an in-app text-based messaging function, with health coaches replying to messages during typical business hours. See Figure 1 for an example interaction between a digital health coach and a patient.

**Table 1:** Example anxiety and depression program techniques.

Technique topic		Technique type	Description
**Anxiety program**			
	Feeling physically tense	Audio-based	Helps the patient relax their muscles by focusing on specific muscle groups.
	Feeling overstimulated	Audio-based	Grounding practice used during emotional distress. The technique asks the patient to observe an object, which they can carry throughout the day.
	Cognitive reframing	Text-based	Helps the patient catch negative cognitive distortions, identify “hot” thoughts, and reframe thoughts for a more balanced alternative.
	Mindfulness	Audio- and text-based	Focuses the patient on slowing down and paying attention to the present moment and teaches the patient to observe feelings and thoughts without any judgment or opinion.
**Depression program**			
	Identifying ABCs	Text-based	Educates the patient on antecedents, behaviors, and consequences and teaches them to connect A, B, and C to one another.
	Feeling sad	Audio-based	Tunes the patient into their 5 senses. The patient chooses a sense to work with by identifying 1 thing they can do to engage that sense.
	Needing a break	Audio-based	The patient imagines a pleasant scene that can have positive physical or mental benefits. Options include a beach, a forest, or a lake.
	Better sleep	Text-based	Educates the patient on sleep and sleep habits, the body clock, and the sleep drive. The patient is given choices for solving specific sleep problems.

### Sample and Data Collection

A convenience sampling approach, across all sites, was used to identify individual providers for participation. Two experienced interviewers (authors KW and BL) conducted semistructured telephone interviews with both BH and PH providers in April–July 2020 to understand facilitators of and challenges in integrating RxWell into their practice as well as their perceptions around sustainability. Interviews were conducted after the 12-week implementation period. Each interview lasted for approximately 30 min. The interview guide (see Multimedia Appendix 1) was developed with input from key stakeholders, including app product designers, a provider, and a qualitative expert.

### Analyses

Interview data were analyzed to identify major themes using an inductive content analysis approach. All interviews were audio-recorded and transcribed verbatim. Transcripts were then reviewed to gain a contextual understanding of the data. Inductive data saturation was assessed to understand whether new themes were described by providers that were not already described in the data [37]. Open coding was conducted on 7 transcripts to allow for initial categorization of relevant themes/concepts. Codes were further created and defined through an iterative process of reviewing 4 additional transcripts, thereby formulating the final global codebook. Dovetail coding software was used for the organization and coding of the data. Two trained independent coders (KW and BL) applied codes to all 19 transcripts. The coders met to review and adjudicate inconsistencies in code application.

## Results

A total of 19 interviews were conducted with health care providers (8 BH providers and 11 PH providers) to discover barriers to and motivators for referring RxWell to their patients. The providers were predominantly 30-49 years old, and most had less than 20 years of clinical practice experience (Table 2). Each interview lasted for approximately 30 min. Four primary thematic categories emerged: benefits and complexities of incorporating the app into the provider’s treatment toolkit, factors that influence both provider and patient engagement, challenges and opportunities with current provider monitoring capabilities, and potential for sustaining app referrals in clinical settings.

**Table 2:** Provider demographics (N=19).

Characteristic		N
**Provider type**		
	PH	11
	BH	8
**Gender**		
	Female	11
	Male	8
**Age (years)**		
	18-29	1
	30-49	11
	50-64	6
	65+	1
**Years in practice**		
	2-9	7
	10-19	8
	20-29	2
	30+	2

### Provider and Patient Engagement Factors

The providers discussed several factors that drive their use/referral of RxWell as a treatment option as well as factors that they believe support patient engagement in the app; often, these factors overlapped. The providers believed they are more apt to trust and refer a patient to RxWell because the content is rooted in a strong, evidence-based treatment theory:

Well, I think it is all organized. It is like giving them the big book of everything that you have in your office where I am constantly compiling stuff like, oh this is good, this is good. And I tell people that talk therapy and CBT and [dialectical behavior therapy] are as good as medicine because that is what I have learned.

BH provider

The providers described how the digital nature of the app allows for greater flexibility for patients to receive education and how it overcomes traditional barriers related to in-person therapy (ie, talk therapy resistance, time, scheduling, travel, and costs). The ability to overcome these barriers was also perceived as a reason why patients would engage with the app:

…As far as having it as a tool to use, I think it is phenomenal because I generally find that people are really reluctant to behavioral therapy. And I know that there is tons of evidence that people with depression and anxiety do better with medication, they do better with behavioral therapy, but they do best when they do both. And yet I cannot get a lot of people to do the behavioral therapy part of it. And I have very few people decline [the app] just because the usual excuses do not work.

PH provider

The providers offered insights into why patients may not engage or stay engaged with the app, including believing that patients needed to have a certain level of motivation and emotional capacity to engage with the app, had a busy lifestyle, or had a lower interest in obtaining support: “The big thing I heard was the motivation factor. Actually, there sometimes would be a desire to use it but they did not actually get over the hump and use it” (BH provider). Motivation was not seen as an app-specific problem, as some providers noted patient motivation can be a challenge with any form of BH treatment.

The providers also commonly mentioned patients forgetting to download the app or speculated that patients forgot to use it. Other barriers to engagement that the providers perceived included patients not owning or feeling comfortable using a smartphone, preferring in-person therapy, and finding provider and app reminders bothersome: “I think it is potentially a little harder for stereotypically older individuals [who] are less tech-savvy individuals who find their phone is merely a communication device rather than an all-in-one type of device” (PH provider).

### Benefits and Complexities of Incorporating the App Into the Provider’s Treatment Toolkit

The providers benefited from having a focused discussion on how to incorporate the app referral process into their existing workflow, including having access to instructional materials. A provider stated, “I very much like to talk things out with people and have those kinds of planning and brainstorming sessions collaboratively…I like being able to walk through the steps of that so I can understand it really well so I can appropriately explain it to someone else” (BH provider). Being able to engage in experiential learning (ie, using the app) was seen as an important experience for providers to partake in during implementation development “because I was able to use it some myself and talk to patients about it from a more personal experience, which is how I like to do therapy. You do such a better job explaining things if you have actually tried it yourself and can really speak to what you liked and did not like” (BH provider). Even providers who did not test the app due to time limitations acknowledged that doing so would have supported their ability to refer patients.

In terms of an addition to usual care services provided, BH and PH providers offered the app to patients for fundamentally different reasons. BH providers approached the app as a supplement to therapy or as a resource that would reinforce, to the patient, what they were already teaching them during in-person therapeutic sessions: “I say, this is a great way to kind of [have] somebody helping you to remember to do skills and that I think it could really add to what they are already getting” (BH provider). PH providers tended to offer the app as 1 of the treatment options available to the patient and often referred patients to the app and provided a medication prescription or a referral to in-person therapy: “I offer CBT on some level to most of my patients with anxiety and depression, and so it becomes an option whether they want to do that or not. Usually I talk about the option of CBT first, and if they are interested in that, then we kind of discuss whether face-to-face or [the app]” (PH provider).

Across provider types, patients were more likely to be referred if they were early in treatment, could use extra educational support, were resistant to other treatment modalities, or were waiting for an initial BH appointment:

I think those barriers [availability and time] are going to increase because we now have issues obviously, with the pandemic…I actually had someone last night that I enrolled who has had a lot of struggles and had kind of relocated to a new area. I do not know that many mental health people are taking new patients right now. He loved the idea that this is something that he could start doing right away to continue kind of with some therapy. And then his intention was still to, because he has got some substance issues, have someone involved. But he was very excited to say, “Okay, I can start this right now. I can do it right now.” Because often there is a week, wait to do it. And then you have kind of lost them that they have sort of past that…I do think that immediacy of it is really important.

PH provider

The providers also shared several reasons for why they would be hesitant or unlikely to refer certain patients to the app, including if the patient was older (eg concern over technology skillset), was happy with their current treatment, preferred in-person therapy, had severe symptoms (eg suicidality, escalating symptoms), or had conditions where the app did not align with treatment goals (eg dysregulation, socially avoidant behaviors):

I have brought it up every time I have thought about it. When I talk to somebody who has depression or anxiety that I think is within reason, people that I think are more severe, I would probably not bring it up as much. If somebody is more of a danger or a threat to themselves, I do not want to. Certainly, in the mild to moderate range, which I would say accounts for 95% of my depression anxiety visits, I am able to bring that up. So those would be the people that I would consider it.

PH provider

Almost all providers described how they typically introduce the app to patients as a treatment option. This initial conversation consisted of a brief overview of the app, often including specific details about the educational components, such as CBT, mindfulness, sleep, and in-the-moment relief options:

At that point, I would describe the app a little bit more and say basically it is not counseling, exactly, that it is an app that teaches them coping mechanisms. Whatever it is that they are going through, it teaches them techniques that help them to better understand what they are going through their emotions and help to work at. I link it a little bit to, a little bit like meditation. They are kind of learning different things, and I just gauge their interest and see if they are interested.

PH provider

Most providers discussed referring and ordering the app for patients during or right after the appointment. BH providers often were able to provide this support: “So, they would get the text [with a link to download the app] and I would have them download it with me and then I would have them go through the process with me of signing up” (BH provider). PH providers were unable to support patients in downloading the app due to time constraints.

### Challenges and Opportunities With Current Monitoring Capabilities

Monitoring patients after referral of the app was an important activity for the providers. Yet many providers were unaware of the in-depth monitoring capabilities (eg GAD-7 and PHQ-8 scores, number of techniques completed, number of messages sent to the digital health coach) within the electronic health record, even though this information was covered during their initial training sessions:

I usually follow up with people; I will have the office staff talk with them within a couple of weeks…so it would be a great additional option to be able to reference part of the chart and see if they were active with something like this. Anything that is objective like that is helpful.

PH provider

The providers commented on key monitoring features that they would like to access directly, including notifications of app progress or non-use, symptom severity alerts, and a population summary of all patients referred to the app, features not currently available in the electronic health record:

I have to say I did not even look at the [electronic health record] report much, but we did get it emailed out to us. I did glance at that and I mean, just seeing the number of referrals and I did think at least 1 of my patients did go through a bunch of activities, so they were at least liking the program. That is helpful encouragement for me to keep using it if I know that my patients are liking it.

PH provider

Further, even though all patients were assigned a digital health coach when they first accessed the app, the providers lacked clarity on the digital health coach role and on how coaches communicated with patients: “I think there was still some lack of clarity about that role exactly, and what that involves, and how people were utilizing that role” (BH provider). However, nearly all providers predicted that it would be helpful for patients to have access to a coach who could support them with tool navigation: “I think that, again, I think it is a great idea. Any kind of relationship like that, even if it is a digital one is certainly, again, another way to maybe connect with someone or just kind of supervise how they are doing, even if it is from afar” (PH provider).

### Sustainability

The providers discussed their ability to see an impact on patients. Although most providers shared that they had not yet received feedback from patients or their patients had not used the app long enough for them to see any impact, there were some indications of early success. In addition, some providers believed the evidence-based principles behind the app would likely yield patient improvements, as traditional CBT has been shown to produce positive impacts: “Well, I do not really know, but I just know overall the skills I am teaching, with the anxiety especially, I see great improvement for the people who want to follow through and learn these things. And I just feel that [the app] will support that” (BH provider). Three providers reported hearing or noticing positive changes in patients and attributed improvements to the app as well as other co-occurring treatments:

…I had 1 young girl. It is interesting because initially when I saw her, and her symptoms were fairly mild and somewhat situational. She really found it was helpful in terms of just checking in with her mood. And then I called her first and she said, “Oh, I have done it some and I really feel good.” We had started meds and then I just did a check in with her last week. And she said, “I have not really done it much, but I know it is there.” She was transitioning and going back to college. She said, “When I get back, I am going to try to do it more.” So, she thought it was helpful and she kind of used it as...In the old days when we used to have people who carry around their Xanax or Klonopin just in case. She sort of felt it was a tool that she had should her symptoms re-escalate.

PH provider

Several providers wanted to understand how use of the app impacted their patients, and mentioned that knowing patient-level outcomes would likely increase their or their patients’ engagement:

I do not [have long-term use concerns], other than I would like to see outcome data with it. But no, I think the barriers [in-person visits/co-pays] to encouraging CBT are the barriers that exist before [the app]. I think it just gives us another option that may be a little bit better for some of our patients.

PH provider

Almost all providers expressed enthusiasm and interest in continuing to refer patients to the app:

I think it would be fantastic. I would love to be able to continue to use modalities like this that are kind of pushed to right where patients are looking to where patients would have the information wherever they are going. They will have it at home, and they will have it while they are waiting in line at a store and they could do it. I think that the downside is pretty low and it is actually, it would be very difficult for me to find significant downsides to it, especially whenever there [are] coaches who are keeping an eye on individuals and we are getting some scores back. So, I think it is all beneficial. I am a big proponent of it.

PH provider

Moreover, considering the COVID-19 pandemic, several providers felt the app is a great option for patients who are not yet able to connect with their therapist via video or do not want to go to in-person provider visits:

I hope that we can continue to use it because there are definitely some patients that I think would have been much harder. I probably would have been more likely to refer some patients out if I did not have [the app] to help things along in between sessions. But I hope that we can continue to use it because people really like it. And I think now that we are adjusting to a world with a lot more telehealth, even figuring out and getting comfortable with how to do that referring in the context of telehealth, I think we have a lot of opportunity there that we are just starting to figure out. So, I would hate to not have it now.

BH provider

## Discussion

### Principal Results

The purpose of this paper was to understand providers’ views on the feasibility and acceptability of delivering RxWell in multiple routine care settings. Overall, we found that providers valued access to RxWell for their patients, believed this tool may serve to overcome traditional barriers related to in-person therapy (ie, talk therapy resistance, time, scheduling, travel, and costs), and viewed having a trained digital health coach support patients in their use of the CBT-based digital app as an added value to care. The providers also shared some hesitancy around prescribing the app to patients with certain characteristics and offered some insights into how to improve patient monitoring.

Although there is an increasing interest among providers to prescribe dCBIs such as RxWell, existing research highlights several concerns and hesitancies among providers around integrating dCBIs into clinical practice, including a lack of integration with clinical workflows; low confidence in the validation and evidence behind app content; concerns over high cost, security, and privacy; and the importance of peer endorsement from other providers [18,28,38]. Some of these concerns can be mitigated through implementation strategies designed to better understand the care settings and contexts [30,39]. Examples of effective strategies to address known challenges include understanding the needs of the target setting and population, engaging relevant stakeholders early in the adaptation and planning process, and obtaining feedback to support an iterative approach [39,40]. As part of this paper, we focused on cocreating workflows through facilitated workshops that served to establish feedback loops between the project team and providers, provide instructional and educational training materials, and identify site-level clinical champions. Our results support these efforts to overcome challenges cited in the literature, as providers believed that engaging in experiential learning (eg, using the app) and having a focused discussion and access to instructional materials are beneficial. They also highlighted the importance of knowing the app content is rooted in evidence and discussing how to incorporate the referral process into their workflow.

Research has also suggested that implementing dCBIs within clinical settings can be better facilitated by establishing organizational support, increasing education and awareness around the apps, and integrating the apps into clinical workflows, such as embedded referrals in the electronic medical record [18,38]. The median visit length for a primary care visit that covers about 6 topics is 15 min [41]. Given these time constraints, it is important to integrate digital app prescription within the provider workflow, and the interviewed providers endorsed the ability to deliver patients RxWell through a 2-click workflow in the electronic medical record and adoption of this digital tool into their daily workflow. However, despite the streamlined workflow, some providers were unable to support patients in downloading the app due to time constraints. Further, the providers discussed not feeling sufficiently educated about how they could monitor user progress within the app and were interested in having a population view of patients they referred, notifications in the electronic medical record related to use or milestones, and patient-level outcome data to support care delivery.

Finally, the providers identified specific factors that influence their decision to refer, including perceptions of the app not working well for patients who are older or patients with severe behavioral symptoms. They were more likely to refer patients who were early in treatment, needed extra educational support, were resistant to other treatment modalities, or were waiting for an initial BH appointment. Although the literature is limited in terms of factors influencing referrals to a dCBI, other studies exploring the decision-making process among providers referring patients to BH solutions have identified comfort with patient diagnoses, the level of familiarity with other BH resources, and patient characteristics as important facilitators of how and when a provider refers a patient to care [41,42].

### Future Implications for Practice

By leveraging the unique position and commitment of an integrated payer-provider health system and its quality improvement efforts to enhance patient and provider experience, scale digital interventions, and ultimately improve health [33], we were able to illuminate key barriers and facilitators from the perspective of providers regarding the referral and use of RxWell. Results from this study will serve as an important early step toward supporting and sustaining provider engagement in digital health. Given our finding that providers are hesitant to refer some patients (ie, patients who are older, are happy with their current treatment, prefer other treatment modalities, or have severe symptoms), future efforts are needed to explore how providers can support both initial and sustained engagement across different patient populations. These efforts may also include strategies such as generating a data-driven list of situations or patient characteristics (eg, geographical locale, technology skill level, severity diagnosis) for which RxWell would be most appropriate, learning more about older adult populations’ challenges in accessing and using digital tools, and continuing to share supportive materials for providers to use as a reference when introducing the app to patients.

The providers also expressed wanting to know more patient-level outcomes among patients they did refer and cited limitations in their capacity to monitor patients over time. Knowing whether a patient has downloaded and used the app, and for how long, offers feedback to the provider about the patient’s engagement in the treatment modality, while information about BH assessment outcomes signals program impact. The ability to scale dCBIs depends upon efforts to make already existing and integrated monitoring functions even more streamlined for providers who have limited time and resources. It is critical to design clinical workflows and referral pathways that meet the needs of the clinical setting and environmental circumstances (ie, COVID-19). Future efforts are needed to explore workflows that provide better visibility into patient-level outcomes, which may increase provider engagement and provide insight into how digital health coaches can further support treatment and therapeutic alliances. Additionally, qualitative evaluations focused on patient and other key stakeholder perspectives are needed to provide a more comprehensive picture of the barriers to and facilitators of successful digital app engagement.

### Limitations

There are several study limitations that are important to acknowledge. Based on our convenience sampling method, results may have limited generalizability due to the nonrepresentative sample. Further, self-selection bias may have influenced the findings and themes, as providers who participated in the interviews may have different perspectives on their experiences referring patients to RxWell than providers who did not respond to the interview invitation. Further, although several interviewee demographics were presented (gender, age, provider type, and years in practice), race/ethnicity data were not collected from interviewees. Finally, the COVID-19 pandemic began amid the study period, which may have impacted provider use of the digital app and uniquely influenced their perceptions. Qualitative interviews illuminated implementation adaptations that providers made during this time, and such adaptations often facilitated the providers’ ability to continue referrals during remote patient visits.

### Conclusions

Our results strongly suggest that providers are supportive of digital tool integration and endorse the use of dCBIs within their workflow. The ability to scale digital interventions to meet the mental health needs of patients relies on streamlined workflows that enable BH and PH providers to easily refer patients to evidence-based interventions. To further enhance provider acceptance of dCBIs, more information is needed about which patients benefit most from such digital tools. Finally, although this paper focuses on factors influencing provider engagement, it is important to gather the perspectives of other critical stakeholders to ensure broad uptake and use of digital interventions that support the BH needs of patients.

## Appendix

Multimedia Appendix 1 Provider interview guide.

## Abbreviations

BH: behavioral health
CBT: cognitive behavioral therapy
dCBI: digital cognitive behavioral intervention
GAD: generalized anxiety disorder
IDFS: integrated delivery and finance system
PCP: primary care provider
PH: physical health
PHQ: Patient Health Questionnaire
