# Supplementary material for: Health Care Provider Perspectives on the Use of a Digital Behavioral Health App to Support Patients: Qualitative Study
Source: JMIR Form Res. 2021 Sep 28;5(9):e28538. doi: 10.2196/28538 (PMC8512194; doi:10.2196/28538)
Supplement: Multimedia Appendix 1 [file formative_v5i9e28538_app1.docx]

**Provider Interview Guide**

1. **Please tell me a little bit about your professional role and the care you provide to patients.**
2. **Thinking back to eight weeks ago, what stands out as being the most beneficial type of support you received while learning about RxWell and how to incorporate it into your workflow?** (Probe about co-design, FAQ sheet for patients, Checklist for Therapists, emails, meeting check-ins, others?)
   1. OK, so now pretend a new provider is onboarding and he/she will be offering RxWell to patients as part of care. What activities, trainings, or information do you feel are critical to making the new provider feel comfortable/able to effectively refer and integrate RxWell into their practice?

Now, I have some general questions about your experience referring and enrolling patients in RxWell.

1. **Could you please walk me though the process of how you refer and enroll a patient into RxWell?** (probe: how they explain RxWell, how they make the referral, how does the download/set-up process work/when it occurs/who supports)
   1. When you introduce RxWell, what aspects of the program seem to be of interest to patients?
   2. When you introduce RxWell, what aspects of the program raise concerns for patients? **(***Probe if concerns ever lead to a patient not wanting to be referred)*
   3. Have there been instances/cases when you decided not to refer patients to RxWell? Please tell me more about that.
   4. What challenges have occurred during the referral and enrollment processes? How did the [insert challenge] get resolved?
   5. Have any of the recent work from home policies related to COVID-19 changed the enrollment process? How so?
   6. Probe on low referrals (if needed)

Next, I would like to ask you about your experiences with the RxWell application and insights patients may have provided you.

1. **What are your general thoughts about RxWell as an additive care tool?**
   1. What do you think are the benefits of integrating RxWell as an additional care tool? Why? *(note: this can be benefits to the providers care provision, and benefits to patients)*
   2. What are some challenges you or your patients have faced related the use of RxWell?
   3. Did you have any patients who were escalated through the RxWell risk protocol? Please tell me about that process.
2. **Do you discuss or bring up RxWell during appointments with patients?** **Why or why not?**
3. Is there any information you would like to know about how to incorporate RxWell into appointments?
4. **As you know, information about what patients have done in RxWell is accessible to you through EPIC via the Digital Care > Monitor tab. What information in the report is most helpful to you?**
   1. Is there any information that was not available in the report that you wish you had?
   2. What would you change about how the information is displayed in EPIC?
5. **Please tell me about your thoughts regarding RxWell Coaches.** *(probe on how the provider interacts with the coach if not mentioned)*
6. **What impact do you think RxWell has had on your patients / their symptoms? Please provide an example.**
   1. What impact do you think RxWell has had on the frequency, duration, or total number of appointments you’ve had with your patients?
   2. Thinking about barriers to mental health care access, what are your thoughts on the usefulness of offering RxWell to patients who are experiencing long wait times to see a mental health provider?
7. **Overall, what are your thoughts about continuing to offer RxWell as an additional tool?**
   1. Do you have any concerns or see any potential barriers related to long-term use of RxWell in your practice?
   2. If time ask: Thinking about how care provision has changed due to new COVID-19 related polices, what are your thoughts about RxWell?
   3. If you could change anything in the RxWell application or coaching process, what would you change? Why?

**Before we finish up, I have a few quick demographic questions for you:**

1. **Provider Type: _____**
2. **Gender: _____**
3. **Age: _____**
4. **Years in Practice: _____**
5. **Anyone else we could speak with**

**Those are all the questions I have for you today. Is there anything that you would like to add? Thanks!**
